# Study on safety and stability of high altitude dump under severe drying-wetting alternation

**DOI:** 10.1371/journal.pone.0273365

**Published:** 2022-08-19

**Authors:** Jianjun Dong, Di Yang, Yuan Mei, Ke Gao

**Affiliations:** 1 College of Safety Science and Engineering, Liaoning Technical University, Huludao, Liaoning, 125000, China; 2 Key Laboratory of Mine Thermo-motive Disaster and Prevention, Ministry of Education, Huludao, Liaoning, 125000, China; Tongji University, CHINA

## Abstract

This study aimed to reveal the impact of the severe drying-wetting process on the safety and stability of high-altitude dumps. Numerical calculations were conducted for the open mining dump of limestone mines for cement in high-altitude mining areas. The distribution law equation of the matric suction and the shear strength equation were imported for unsaturated soil based on the unsaturated-saturated seepage theory. Therefore, the evolution characteristics of the unsaturated-saturated seepage field and the stability of the dump were studied under severe drying-wetting. The results indicated the following rules: As the intensity of the wetting-drying alternation increased, the surface soil on the dump changed from an unsaturated to a saturated state, the matric suction continued to decrease until it reached zero, the shear strength decreased, and the unsaturated area shrank. The dump slipped under the influence of the drying-wetting alternation, the sliding area was the dump itself, and developed to the deep layer as the intensity of the drying-wetting alternation strengthened. The cumulative settlement value of the dump increased with time and eventually stabilized, and the maximum accumulative settlement value calculated by simulation was in good agreement with the actual monitoring value. The safety factor decreased as the intensity of the drying-wetting alternation increased.

## Introduction

There is a unique plateau monsoon, which is formed by hot low-pressure in summer and cold high-pressure in winter. The dry season is controlled by the west winds in the winter half year, whereas the wet season is controlled by the southwest and southeast monsoons in the summer half year. The precipitation is concentrated in the summer half of the year, resulting in the phenomenon of evident and severe alternation of wet and dry seasons [1]. As the dump is exposed to the drying-wetting alternation environment for long periods, the shear strength of the soil reduces, which eventually leads to landslides. Therefore, studying the drying-wetting alternation phenomenon caused by rainfall has a considerable engineering significance for the stability of high-altitude dumps.

Feng et al. used indoor uniaxial tensile tests to analyze the law of tensile strength of collapsing soil under the influence of drying-wetting cycles and concluded that when the number of drying-wetting cycles increased, the tensile strength gradually attenuated and finally stabilized [2]. Cai et al. studied the failure law of expansive soil channel slopes through centrifugal simulation experiments and revealed that the canal slope forms the phenomenon of repeated drying-wetting alternation due to repeated water supply and shut-off. The slope soil cracks gradually expand and the canal slope is shallowly destabilized and damaged [3]. Qi et al. studied the change in the shear strength of red clay under different drying-wetting cycles and concluded that the shear strength of red clay decreased as the number of drying-wetting cycles increased, and the degree of attenuation gradually decreased [4]. Liu et al. conducted direct shear tests on red sandstone joints under drying-wetting cycles to study the influence of these cycles on the shear mechanical properties of a jointed rock mass. The results indicated that the internal friction angle and cohesion constantly decreased as the number of drying-wetting cycles increased, and the cohesion decreased more significantly [5]. Yu et al. studied the stability of granite residual soil slope by numerical simulation and direct shear tests and found that with the increase of the number of drying-wetting cycles and the cycle strength, the shear strength of the slope decreases, and the shear strength remains basically unchanged after 3–5 dry-wet cycles. And they obtained the shear strength formula of granite residual soil under drying-wetting cycle condition by data analysis [6]. De et al. established a numerical model for cutting slopes to study the strength change of granite residual soil under a drying-wetting cycle. The results indicated that the drying-wetting cycles affected the stability of the granite residual soil cutting slope, decreasing the slope stability by 10–30% [7]. Yuan et al. studied the changes in the permeability characteristics of compacted loess under wetting-drying cycles. For compacted loess with the same dry density, they observed that as the number of drying-wetting cycles increased, the permeability of the compacted loess improved. However, under the same number of wetting and drying cycles, the permeability showed an opposite tendency [8].

The above studies mostly focused on the impact of rainwater infiltration or conventional drying-wetting alternation on the stability of low-altitude slopes, and the meaning of the severe drying-wetting alternation was a process of alternate transformation between the two extreme states of the unsaturated soil, that was, the alternation of the residual water content and the saturated water content. For example, the drying-wetting alternation under extreme rainfall means that the unsaturated soil undergoes a strong hygroscopic process in which the residual water content was converted to the saturated water content with high hydraulic gradient. Slopes are more prone to landslides in this environment.

Therefore, this study focused on the final dumping site of the cement limestone mining area in the Mum mining area, Sangri County, Tibet Autonomous Region. Based on the experimental data and unsaturated soil shear strength formula, a numerical calculation model for the stability of dumps under severe drying-wetting alternation was established. Moreover, the variation of the unsaturated-saturated seepage field and the stability of the dump under different alternating wet and dry intensities provided a reference basis for the slope landslide disaster of the open mining dump in the high-altitude mining area.

## Fundamental

### Shear strength theory of unsaturated soils

Vanapelli et al. [9] developed an equation for the shear strength of unsaturated soils:

$$\tau_{f}=[c^{\prime }+(\sigma_{n}-u_{a})\tan\phi^{\prime }]+(u_{a}-u_{w})(\theta /\theta_{s}\tan\phi^{\prime })$$ (1)

where *τ*_*f*_ is the shear stress on the failure surface when the soil fails (kPa), *c*′ is the effective cohesion (kPa), *σ*_*n*_ is the normal stress (kPa), *u*_*a*_ is the pore gas pressure (kPa), *ϕ*′ is the effective internal friction angle (°), *u*_w_ is the porewater pressure (kPa), *θ* is the volumetric water content, and *θ*_*s*_ is the saturated volumetric water content.

The following formula can be obtained by transforming:

$$\tau_{f}=[c^{\prime }+(\sigma_{n}-u_{a})\tan\phi^{\prime }]+(u_{a}-u_{w})(\frac{S-S_{r}}{1-S_{r}}\tan\phi^{\prime })$$ (2)

where *S* is the saturation, *S*_*r*_ is the residual saturation, and the effective saturation is *S*_*e*_ = (*S*−*S*_*r*_)/(1−*S*_*r*_).

Therefore the unsaturated soil shear strength formula is given as follows:

$$\tau_{f}=[c^{'}+(\sigma_{n}-u_{a})\tan\phi^{'}]+S_{e}(u_{a}-u_{w})\tan\phi^{'}$$ (3)

When u_a_ = u_w_, Eq (3) is the saturated shear strength formula [10,11]. In light of Eq (3), the expression for total cohesion is defined as [12,13]:

$$c_{t}=c^{\prime }+S_{e}(u_{a}-u_{w})\tan\phi '$$ (4)

where *c*_*t*_ is the total cohesion (kPa), not the total stress cohesion.

### Seepage theory for unsaturated soils

The soil unsaturated-saturated seepage equation [14] is expressed in tensor form as:

$$q_{i}=-k_{r}(S)K_{ij}h_{,}{}_{j}=k_{r}(S)K_{ij}{\left[\psi +z\right]}_{,j}$$ (5)

where *q*_*i*_ is the unit flow vector, *K*_*ij*_ is the permeability coefficient tensor, and *K*_*r*_(*S*) is the relative permeability coefficient. When the area is saturated, *K*_*r*_(*S*) = 1; when the area is unsaturated, 0<*K*_*r*_(*S*)<1; *h*_*j*_ is the hydraulic gradient; *ψ* is the pressure head, *ψ* = *u*_*w*_/*γ*_*w*_ (kPa); *γ*_*w*_ is the unit weight of water (kN/m^3^); and z is the position head (kPa).

Eq (5) shows that when saturation is 1, the saturated seepage of the soil is unsaturated. To realize unsaturated seepage in soil, the most important aspect is to establish a functional relationship between the unsaturated permeability coefficient and saturation. Soil matric suction appears as a negative pore water pressure in the natural state.

The Van Genuchten (VG)–Mualem model is a combination of the soil-water characteristic curve (SWCC) model and permeability coefficient models [15,16]. The corresponding equation is:

$$k_{u}=kS_{e}^{0.5}{\left[1-{\left(1-S_{e}^{1/m}\right)}^{m}\right]}^{2}$$ (6)

The equation of the VG model is:

$$S_{e}=S-S_{r}/1-S_{r}=(\theta -\theta_{r})/(\theta_{s}-\theta_{r})={\left\{1/[1+{\left(\alpha s\right)}^{n}]\right\}}^{m}$$ (7)

where *k*_*u*_ is the unsaturated permeability coefficient (m/s); *k* is the saturated permeability coefficient (m/s); *n*, *α*, and *m* are fitting parameters of the VG-holding water model in the matric suction (kPa). In the numerical calculation, the construction of the unsaturated soil seepage model must include the saturation parameter in the governing, motion, and constitutive equations, and the SWCC must be defined at the same time.

### Distribution law of the matric suction

In the natural state, the vertical distribution law of the matric suction of unsaturated soils is related to the depth, and the vertical unsaturated flow in the steady state conforms to Darcy’s law, which is given by:

$$q=-k_{u}(d(u_{a}-u_{w})/\gamma_{w}dz+1)$$ (8)

The unsaturated permeability coefficient is a function of the matric suction, and it can be solved using Gardner’s theoretical model [17] for the characteristic parameters of unsaturated soil seepage [18]:

$$k_{u}=k\exp[-\beta (u_{a}-u_{w})]$$ (9)

where *β* is the rate of change of the soil permeability coefficient dependent on the suction of the matrix (kPa^-1^).

Combining Eqs (8) and (9), the following equation is derived:

$$q=-k\exp[-\beta \psi_{h}\gamma_{w}][d\psi_{h}/dz+1]$$ (10)

where *ψ*_*h*_ is the suction head, *ψ*_*h*_ = (*u*_*a*_−*u*_w_)/*γ*_w_.

Integrating Eq (10), when the boundary condition is z = 0, the suction force can be obtained as:

$$u_{a}-u_{w}=(-1/\beta )\ln[(1+q/k)\exp(-\beta z\gamma_{w})-q/k]$$ (11)

When the hydrostatic pressure condition is q = 0, the suction is linearly distributed as:

$$u_{a}-u_{w}=z\gamma_{w}$$ (12)

### Calculation method of safety factor

In the numerical calculation, the “disection method” is used to solve the safety factor [19,20], and the expression of the strength reduction method is:

$$c_{F}=c_{t}/F_{r}=[c^{\prime }+s_{e}(u_{a}-u_{w})\tan\varphi^{\prime }]/F_{r}$$ (13)

$$\varphi_{F}=\tan^{-1}(\tan\varphi^{\prime }/F_{r})$$ (14)

where *c*_*F*_ is the reduced cohesion (kPa), *φ*_*F*_ is the reduced internal friction angle (°), and *F*_*r*_ is the reduction coefficient.

The expression for the safety factor of the strength reduction method for unsaturated-saturated soils is:

$$K=\int_{0}^{1}\left(c^{\prime }+[\sigma_{n}-u_{a}+s_{e}(u_{a}-u_{w})\tan\varphi^{\prime }]\right)dl/\int_{0}^{1}\tau dl$$ (15)

Based on the above theoretical system, a numerical calculation program was developed and written on the FLAC3D platform using Fish.

## Numerical analysis

### Engineering overview

**Fig 1:**
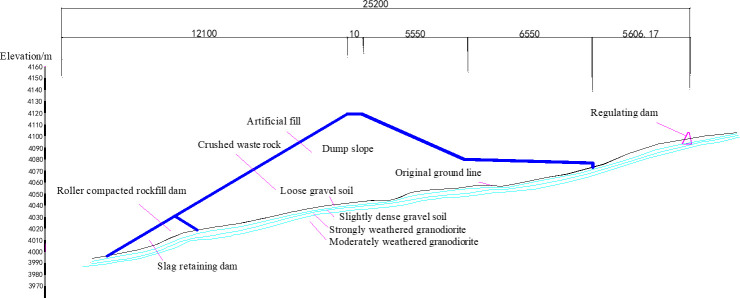
Profile of limestone dump.

The average altitude of the cement limestone mining area in the Mamu mining area, Sangri County, Tibet Autonomous Region, is over 4000 m, which is a typical high-altitude area.The dump is in a valley terrain, and the stratum distribution of the dump from the surface to the bottom is as follows. (1) Quaternary artificial accumulation layer: mainly artificial fill, consisting of silty clay sandwiched within abandoned limestone blocks. (2) Quaternary alluvial strata: mainly composed of gravel soil, which is divided into loose and slightly dense gravel soils. The gravel content in the loose gravel was approximately 50–55%, the particle size was mostly 2–12 cm, and a small amount was larger than 12 cm. The composition was quartz sandstone, granodiorite. (3) Early Cretaceous granodiorite: Its lithology was mainly granodiorite, which could be divided according to the degree of weathering into strongly and moderately weathered granodiorites. Strongly weathered granodiorite is blue-gray and gray-white, mainly composed of minerals such as quartz, feldspar, and mica. It has a coarse-grained structure, massive structure, local diorite grains, and developed weathered fissures in the strongly weathered zone. The core is mostly fragmented and the rock is soft. The core quality designation (RQD) is 0. The thickness of the rock formation is about 1.5m-3.2m. The stratum distribution of the dump is shown in Fig 1.

### Model building and parameter determination

**Fig 2:**
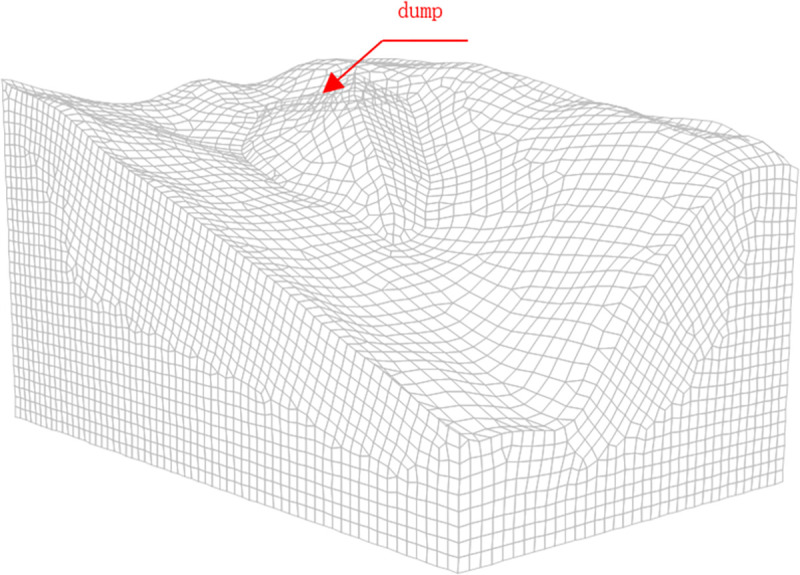
Model diagram.

The research object was a slope with the maximum dumping volume at the end of the dumping site. Based on the finite-difference numerical calculation platform FLAC3D, the calculation program was compiled with FISH language, and numerical calculations and analyses were conducted. The slop-dump model is shown in Fig 2. The size of the established geometric model was 356.4 m × 611.4 m ×335.7 m, and the geometric model was divided into 60,588 units and 47,792 nodes.

Mechanical boundary conditions: Fixed constrained boundaries existed around the model and at the bottom, and the upper part was a free boundary. Seepage boundary conditions: The upper surface of the model was set as the permeable boundary, and the surrounding area of the model was set as an impermeable boundary. The Mohr–Coulomb elastic-plastic model was used for the soil constitutive model, and the isotropic seepage model was used for the soil in the seepage calculation.

**Fig 3:**
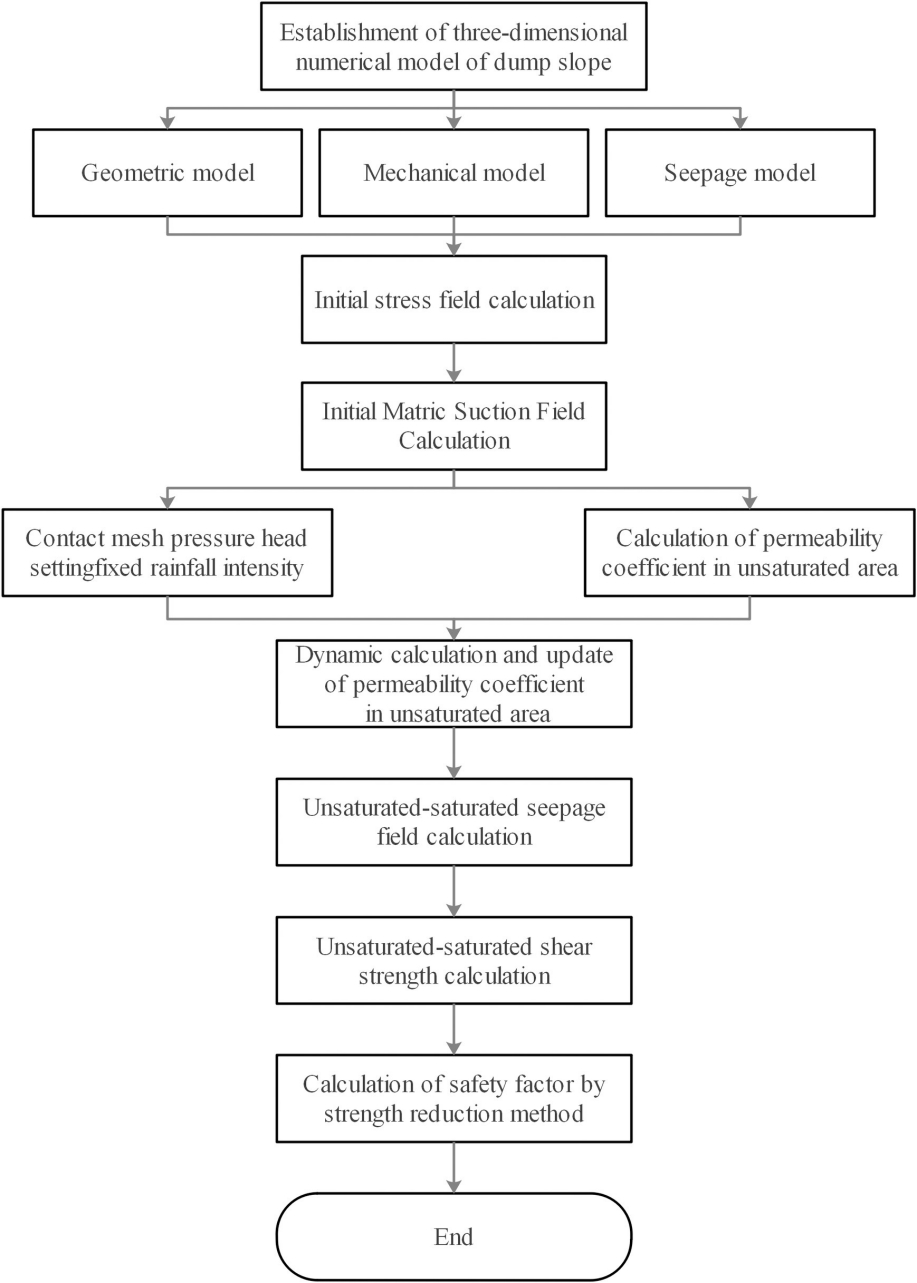
The calculation process of severe drying-wetting alternation.

In order to simulate the calculation of severe drying-wetting alternation, it was necessary to use Fish language for secondary development, and the focus of secondary development was that the model can be used for unsaturated-saturated seepage calculation, dynamic update of unsaturated zone permeability coefficient, unsaturated-saturated shear resistance Intensity calculation, and then realized the calculation of severe drying-wetting alternation, the specific calculation process was shown in Fig 3.

#### (1) The shear tests

**Fig 4:**
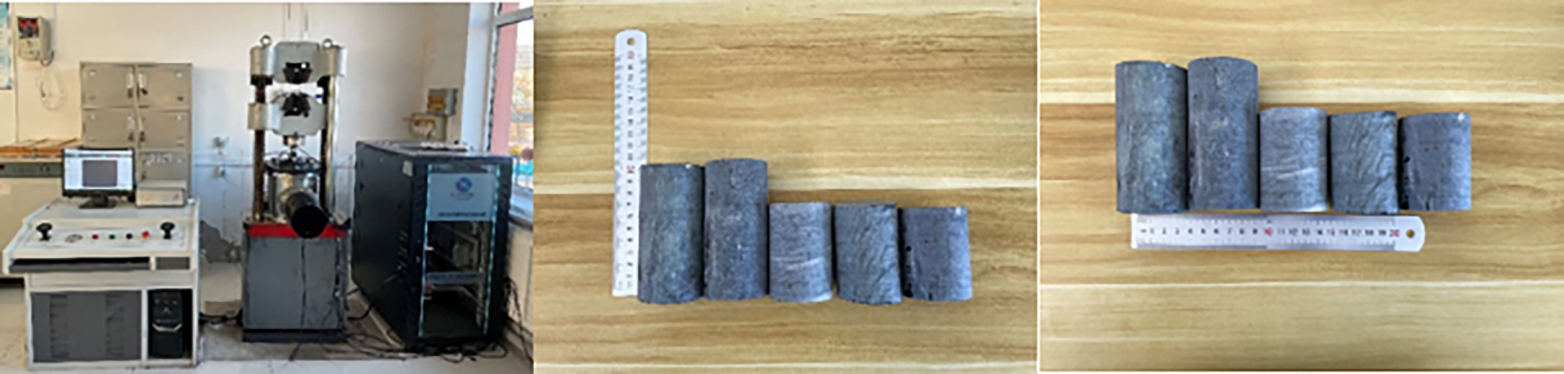
Experimental equipment.

According to the geological exploration, there are 7 types of geotechnical materials in the dump slope, namely roller compacted rockfill dam, artificial fill, crushed waste rock, loose gravel soil, slightly dense gravel soil, strongly weathered granodiorite and moderately weathered granodiorite. Roller compacted rockfill dam, artificial fill, and crushed waste rock are randomly sampled on the dump slope. Loose gravel soil, slightly dense gravel soil, strongly weathered granodiorite and moderately weathered granodiorite are obtained during geological exploration. 5 samples were taken for each type of geotechnical material, and a total of 35 samples were obtained. The test equipment and rock samples are shown in Fig 4, and the test results are presented in Table 1.

**Table 1:** Mechanical parameters of soil in dump.

Engineering materials	Natural test weight (kN/m^3^)	Saturated bulk density (kN/m^3^)	Friction angle (°)	Underwater friction angle (°)	Cohesion (kPa)	Underwater cohesion (kPa)
Roller compacted rockfill dam	20.5	–	35	–	5	–
Artificial fill	21.5	22.5	25.3	23.7	4	3
Loose gravel soil	20.5	21.0	20	18	8	4
Slightly dense gravel soil	21.0	22.0	22	20	10	5
Strongly weathered granodiorite	22.5	22.8	30	25	40	30
Moderately weathered granodiorite	25.2	25.5	38	35	500	400
Crushed waste rock	20.4	–	35	33	–	–

#### (2) The SWCC tests

**Fig 5:**
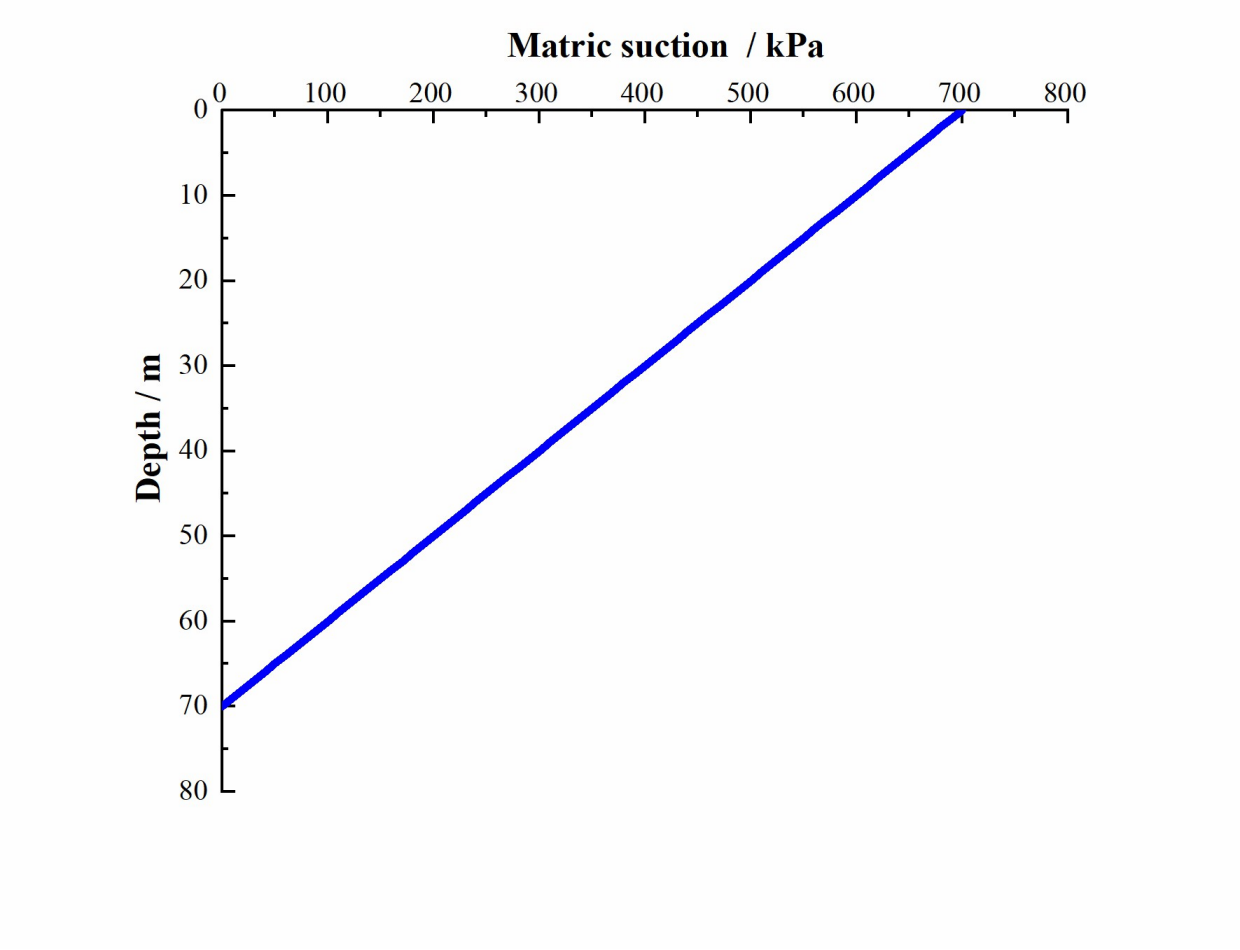
Suction distribution law of unsaturated soil.

Through on-site investigation, it was determined that the buried depth of the groundwater table was 70m, and the suction force of the soil at the location of the groundwater table was 0kPa. Therefore, according to Eq (12), the suction value of the surface was calculated to be 700kPa, and the magnitude of the matric suction was affected by the depth of the soil. As the depth of the soil increased, the matric suction decreased linearly, as shown in Fig 5.

According to Eq (7), the relationship between the matric suction and the volumetric water content could be obtained as:

$$\theta =(\theta_{s}-\theta_{r}){\left[\frac{1}{1+{\left(\alpha s\right]}^{n}}\right]}^{m}+\theta_{r}$$ (16)

**Fig 6:**
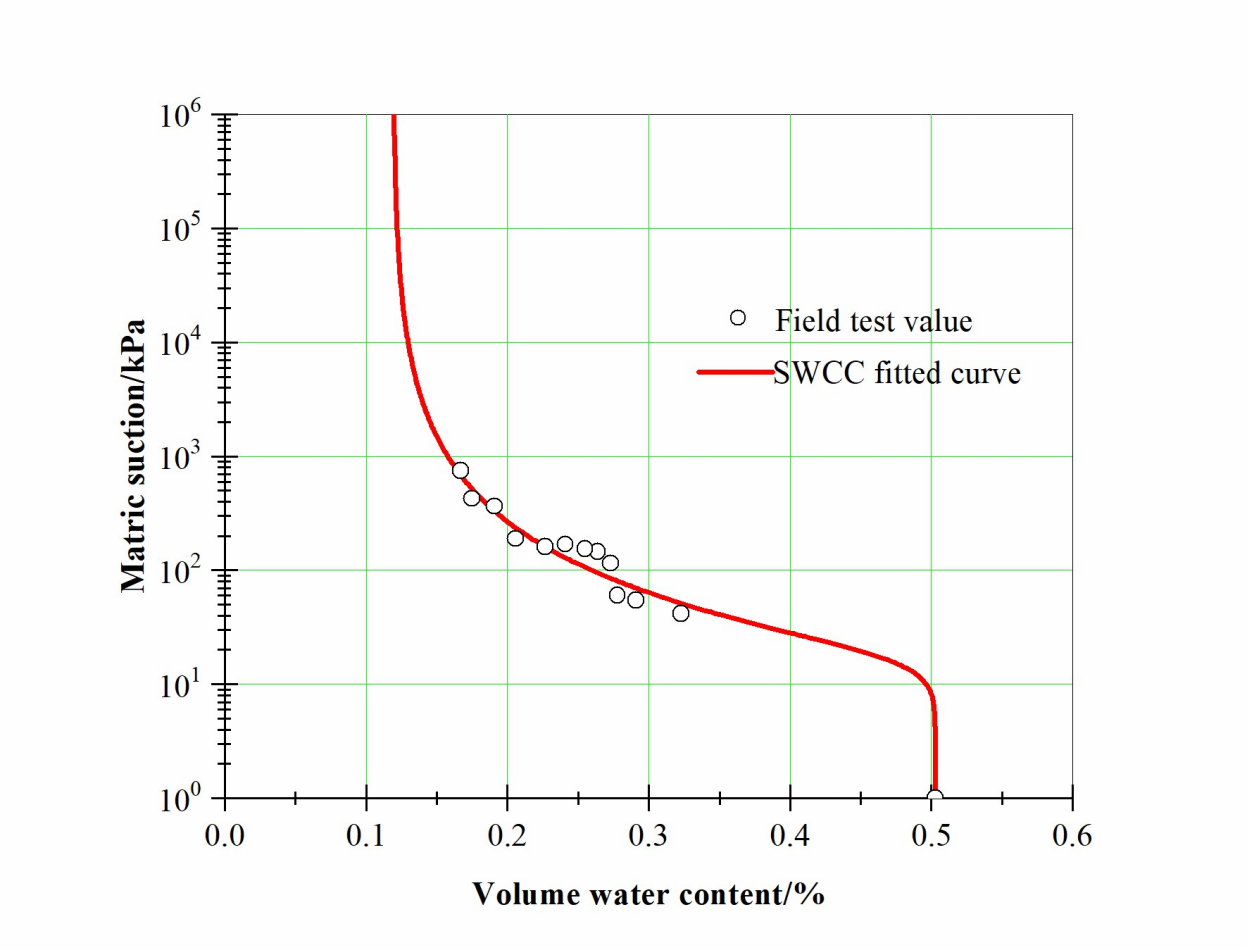
Soil—water characteristic curve.

According to the distribution law of the matric suction, based on the water content test data and Eq (15), the soil-water characteristic curve was fitted to obtain the SWCC fitting parameters. The fitting results are shown in Fig 6.

Using the method of combining the experimental data with the empirical model, the model parameters could be solved under the condition of limited experimental data. The degree of agreement between the experimental value and the calculated curve could also be calibrated. At the same time, this method makes up for the inability to obtain a complete soil-water characteristic curve (SWCC) due to incomplete data. And the basic parameters of the SWCC curve model expression were determined by analyzing the experimental data, as shown in Table 2.

**Table 2:** Basic parameters of SWCC for dump model.

*β* (kPa^-1^)	*k*_*s*_ (m∙s^−1^)	*γ*_*w*_ (kN⋅m^-3^)	*θ*_*s*_ (%)	*θ*_*r*_ (%)	*α*	*m*	*n*
0.005	2.5×10^−6^	9.8	50.29	11.90	0.06	0.15	4

### Seepage parameters selection

**Fig 7:**
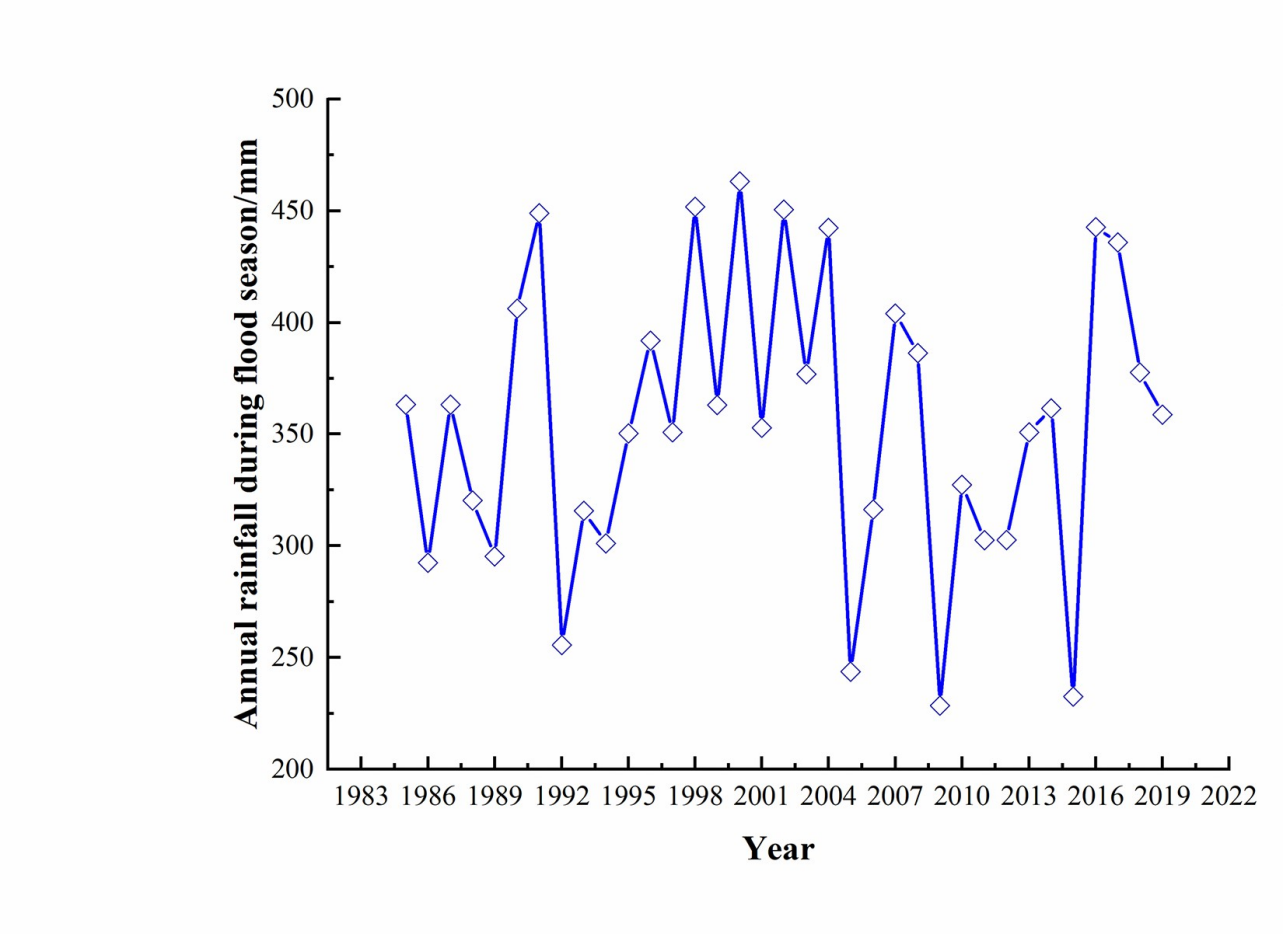
Variation of annual rainfall in flood season.

When the slope of the dump and the surrounding mountains were subjected to rainfall, statistics were collected on the meteorological data of Shannan City, Tibet, and the tendency map of annual rainfall during the flood season over the past 30 years was analyzed (Fig 7). In Shannan City, the maximum precipitation in the rainy season over the past 30 years was approximately 56.7 mm in 3h, and the rainfall intensity was 5.25×10^−6^ m/s.

### Result analysis

#### (1) Analysis of unsaturated-saturated seepage results

**Fig 8:**
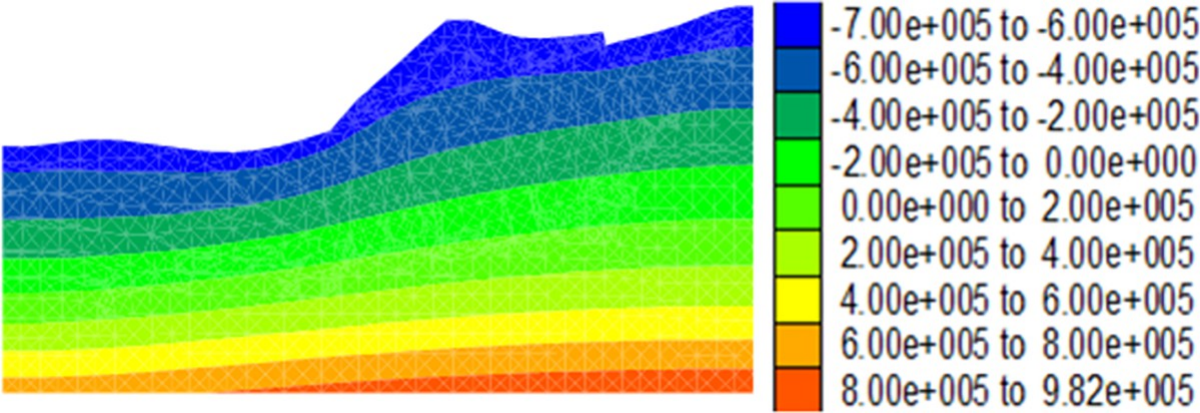
Pore water pressure cloud map in the dry season of the dump (unit: Pa).

During the alternating drying-wetting seasons, the rainfall intensity of the first rainfall reflected the drying-wetting alternation intensity. The first precipitation *Q*_1_ in the alternation of drying-wetting seasons reflected the intensity of drying-wetting alternation in Shannan, where *Q*_1_ < 113.4 mm, 113.4 mm ≤ *Q*_1_ ≤ 226.8 mm, and *Q*_1_ > 226.8 mm represent low-, medium-, and high-intensity drying-wetting alternations, respectively. The distribution of porewater pressure on the dump in the dry season is shown in Fig 8. The zero value line of the pore water pressure corresponds to the groundwater level line. The pore water pressure of the unsaturated soil above the groundwater level was negative, and the pore water pressure of the saturated soil below the groundwater level was positive.

**Fig 9:**
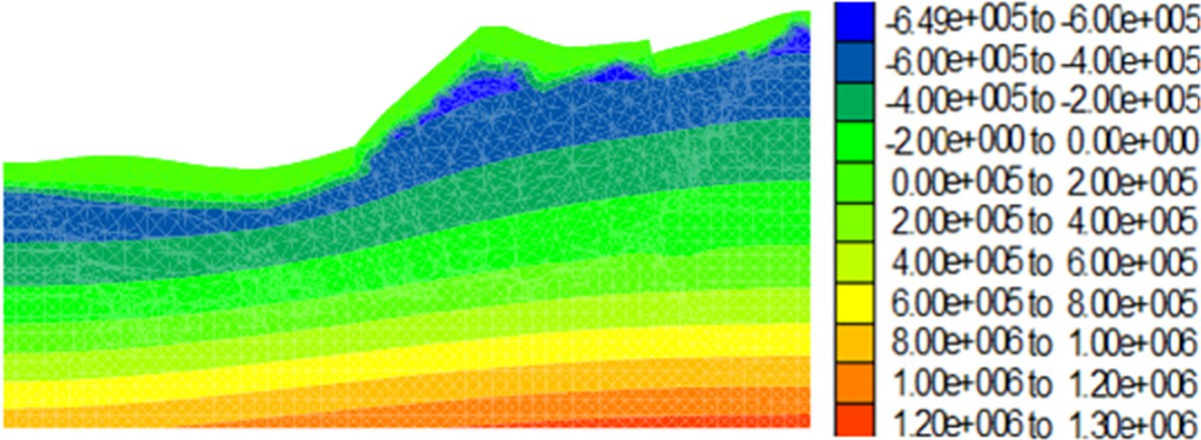
Diagram of pore pressure variation of the dump when the drying-wetting alternate intensity is 56.7mm (unit: Pa).

**Fig 10:**
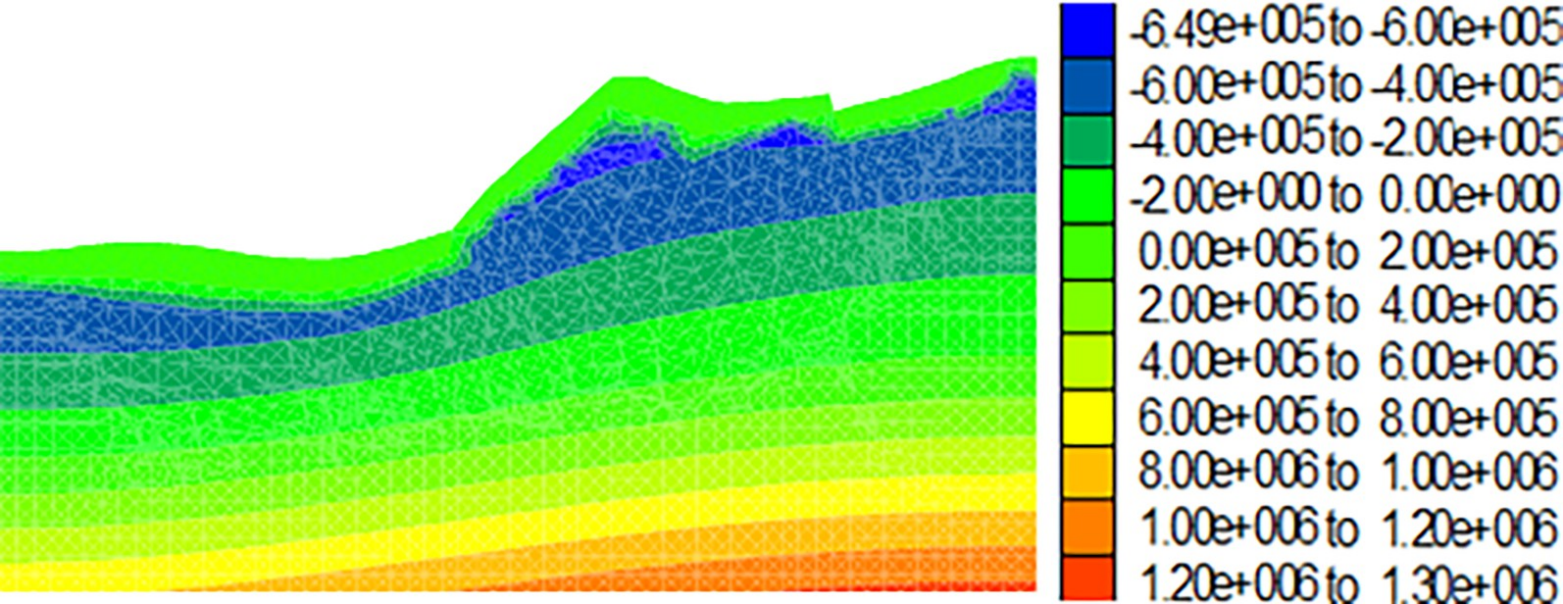
Diagram of pore pressure variation of the dump when the drying-wetting alternate intensity is 113.4mm (unit: Pa).

**Fig 11:**
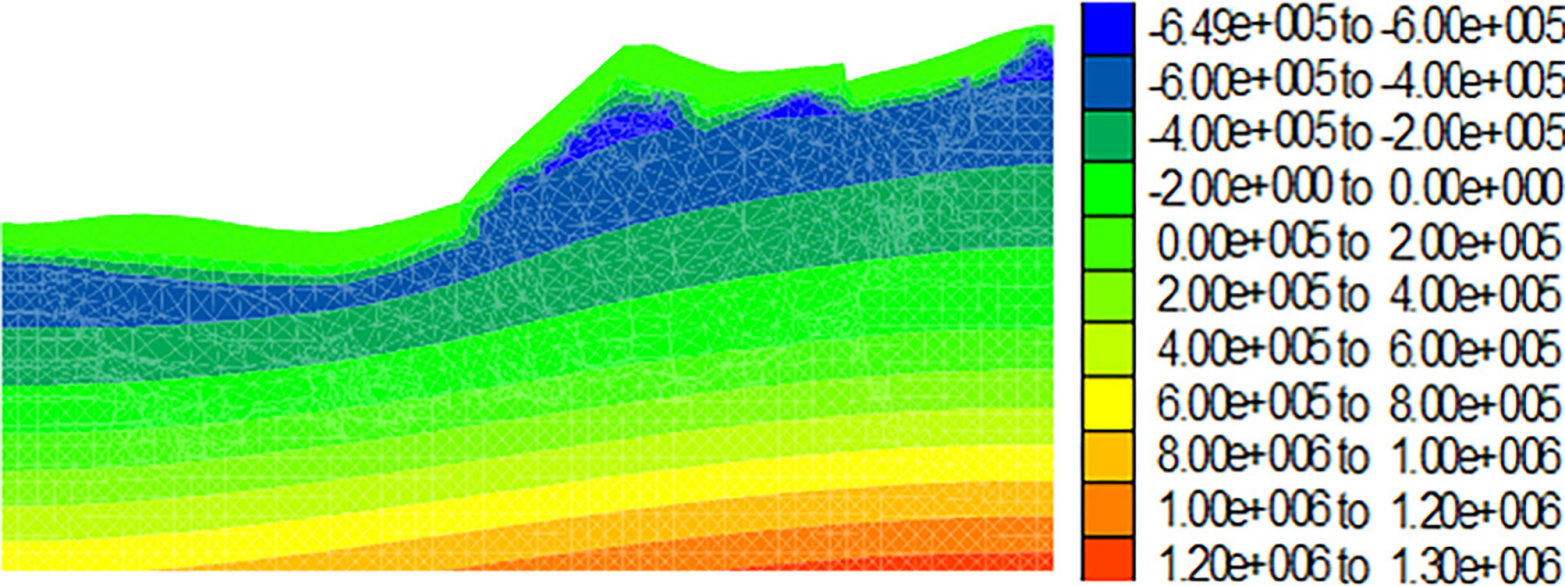
Diagram of pore pressure variation of the dump when the drying-wetting alternate intensity is 226.8mm (unit: Pa).

**Fig 12:**
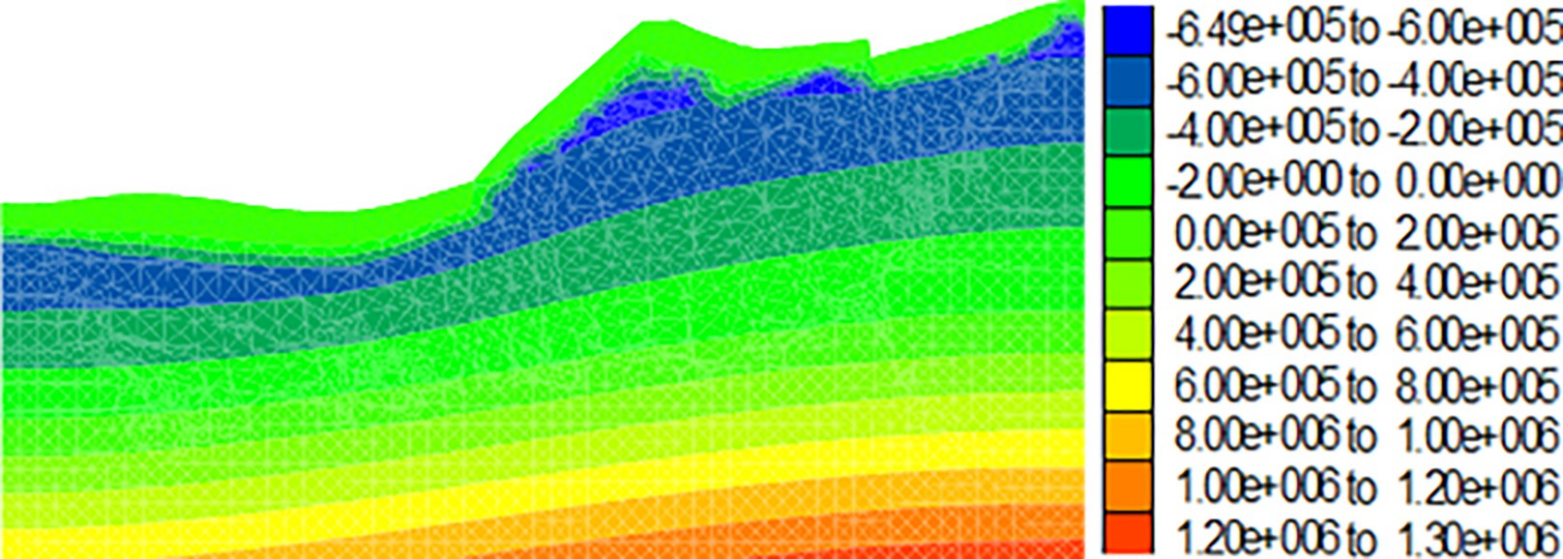
Diagram of pore pressure variation of the dump when the drying-wetting alternate intensity is 453.6mm (unit: Pa).

Figs 9–12 show the calculation results for the dump after the drying-wetting alternation. By comparing Figs 8 and 9–12, With the increase of the drying-wetting alternation strength *Q*_1_, the pore water pressure value of the shallow slope body and the shallow mountain body increased from -700kPa to 0kPa, the unsaturated area was also reduced, and the pore water pressure below the wetting fraction was redistributed according to the gradient. This indicated that the shallow slope body of dump slope had transformed from an unsaturated state to a saturated state, that is, the matric suction of the shallow slope of the dump slope was 0, and a transient saturation region appeared. At the same time, the shear strength of the dump slope also decreased with the increase of the drying-wetting alternation strength, which affected the stability of the dump slope.

#### (2) Variation in the slip area of the dump under alternating drying-wetting conditions

**Fig 13:**
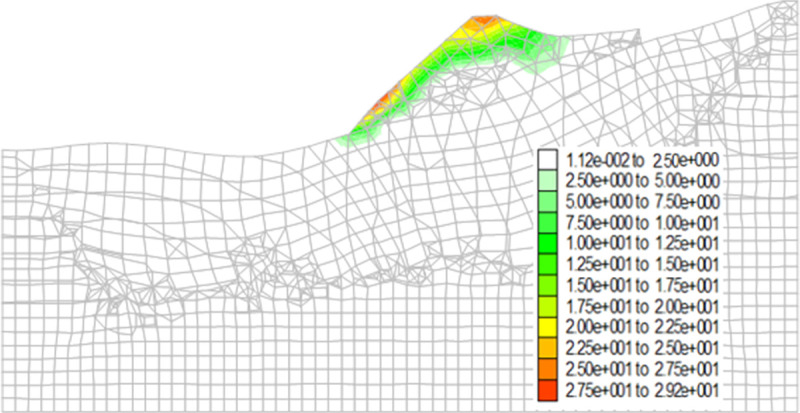
Shear strain increment of the dump when the drying-wetting alternate intensity is 56.7mm.

**Fig 14:**
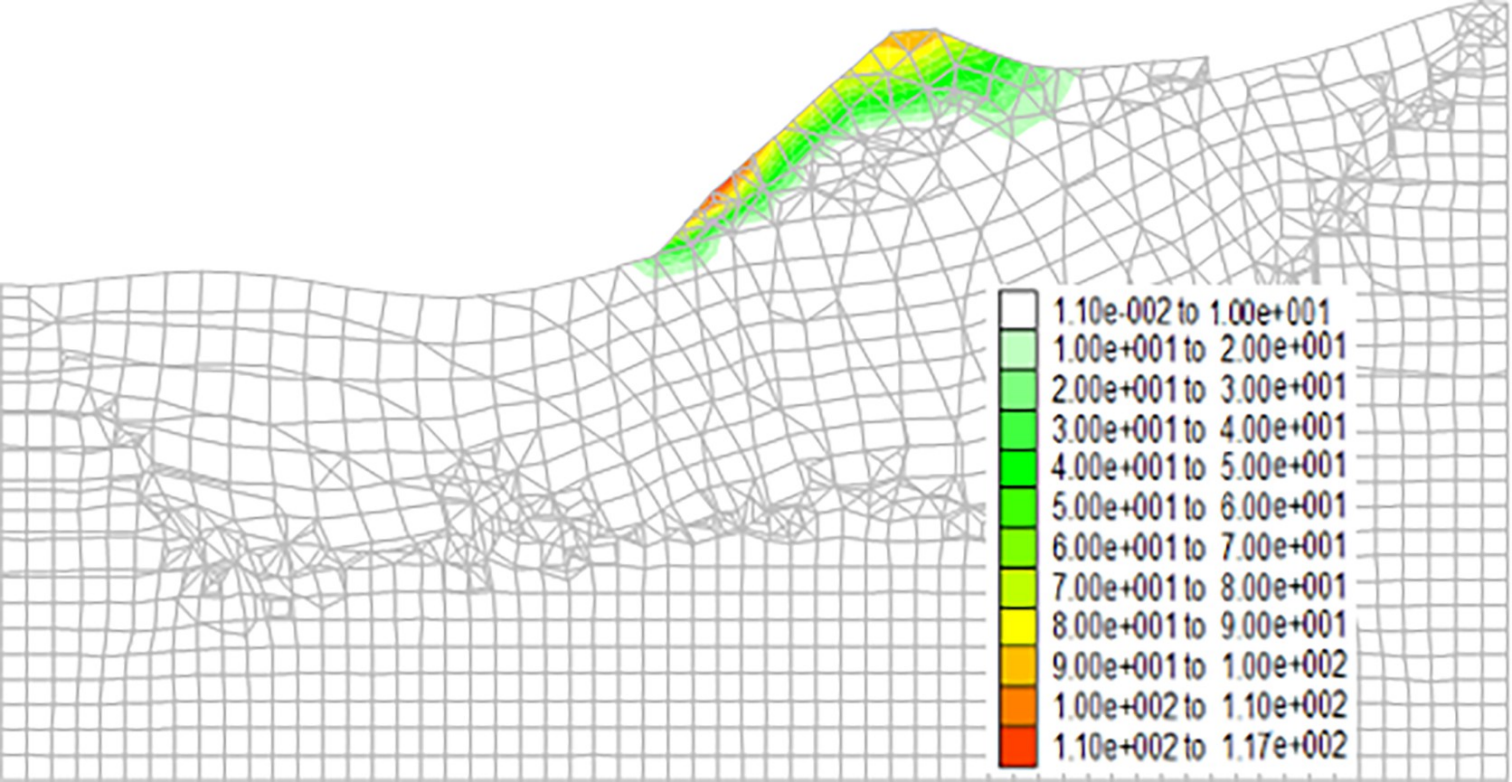
Shear strain increment of the dump when the drying-wetting alternate intensity is 113.4mm.

**Fig 15:**
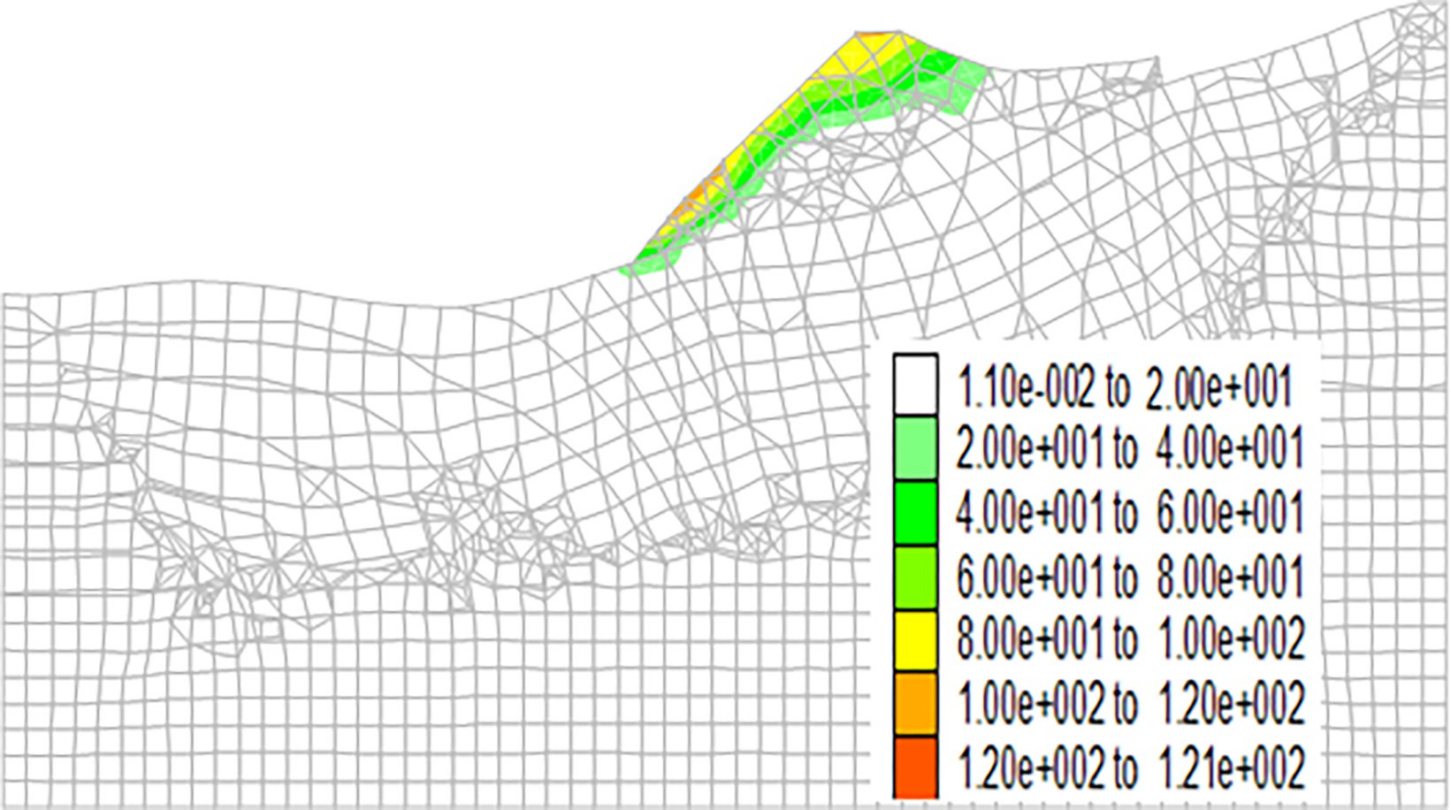
Shear strain increment of the dump when the drying-wetting alternate intensity is 226.8mm.

**Fig 16:**
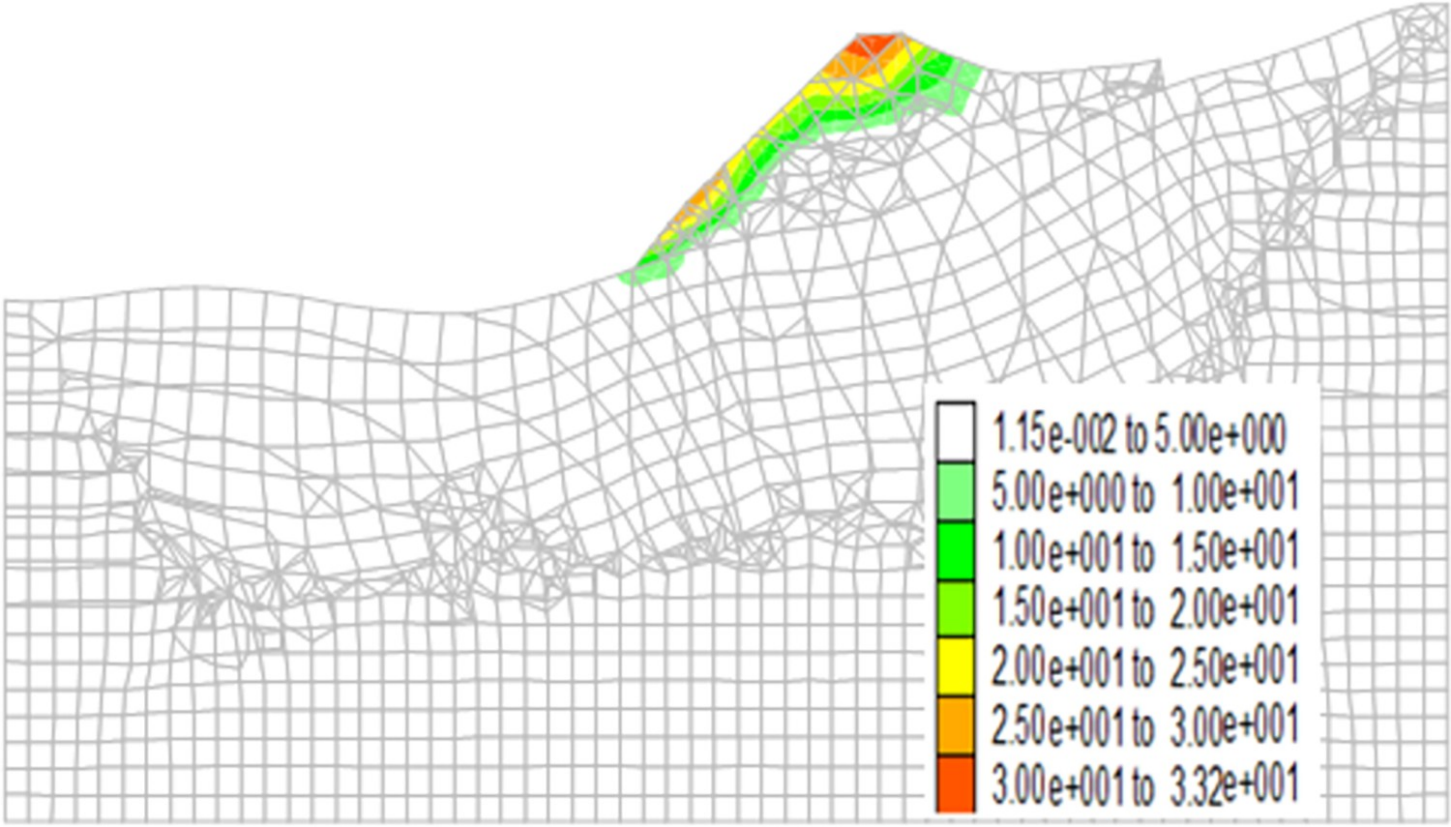
Shear strain increment of the dump when the drying-wetting alternate intensity is 453.6mm.

Figs 13–16 show the cloud maps of the shear strain increment on the dump under different drying-wetting alternation intensities.

As shown in Figs 13–16, the first shear failure area of the dump slope is the shallow slope body at the top and toe of the slope. As the intensity of drying-wetting alternation increases, the shear failure area gradually develops to the deep slope body. The sliding area is located in the dump slope, which indicates that the dump is in a dangerous state under the influence of severe drying-wetting alternation.

#### (3) Variation in the cumulative maximum settlement value of the dump under different drying-wetting alternation intensities

**Fig 17:**
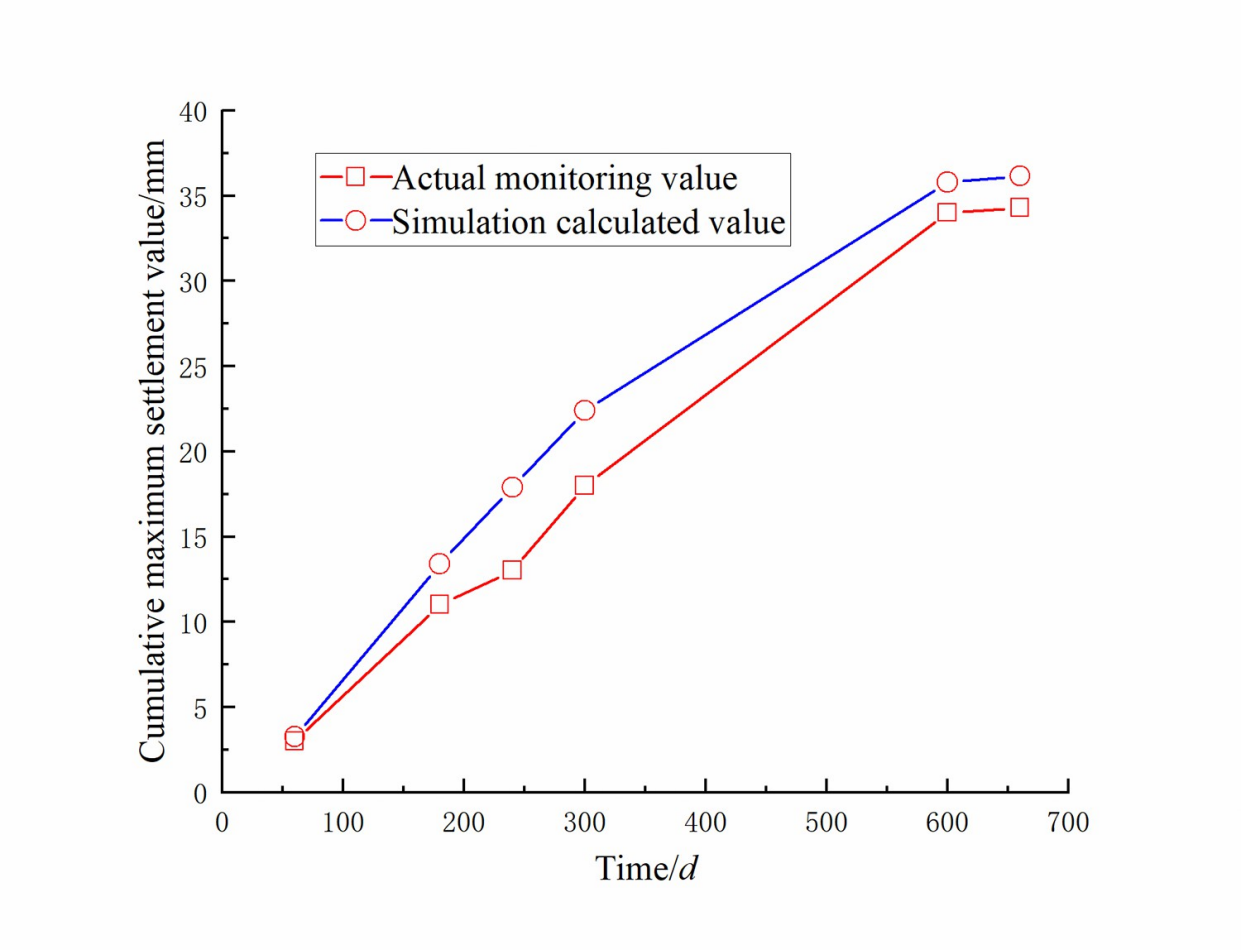
Maximum accumulated settlement value *Z*_*max*_ of the dump under different drying-wetting alternating intensities.

Monitoring the settlement value of the landfill from November 4, 2018 to December 23, 2020 was shown in Fig 17. The red curve in Fig 17 was the actual monitored maximum accumulated settlement value of the dump slope, and the blue curve was the maximum cumulative settlement value of the dump slope obtained by simulation calculation. It could be seen from Fig 17 that the trend of the actual monitored cumulative maximum settlement value and the cumulative maximum settlement value obtained by the simulation calculation first gradually increased from 2018/11/04 to 2020/12/23, and then tended to be flat. It could also be observed that the actual monitored maximum cumulative settlement was in good agreement with the simulated maximum cumulative settlement.

#### (4) Safety factor of the dump under different drying-wetting alternation intensities

**Fig 18:**
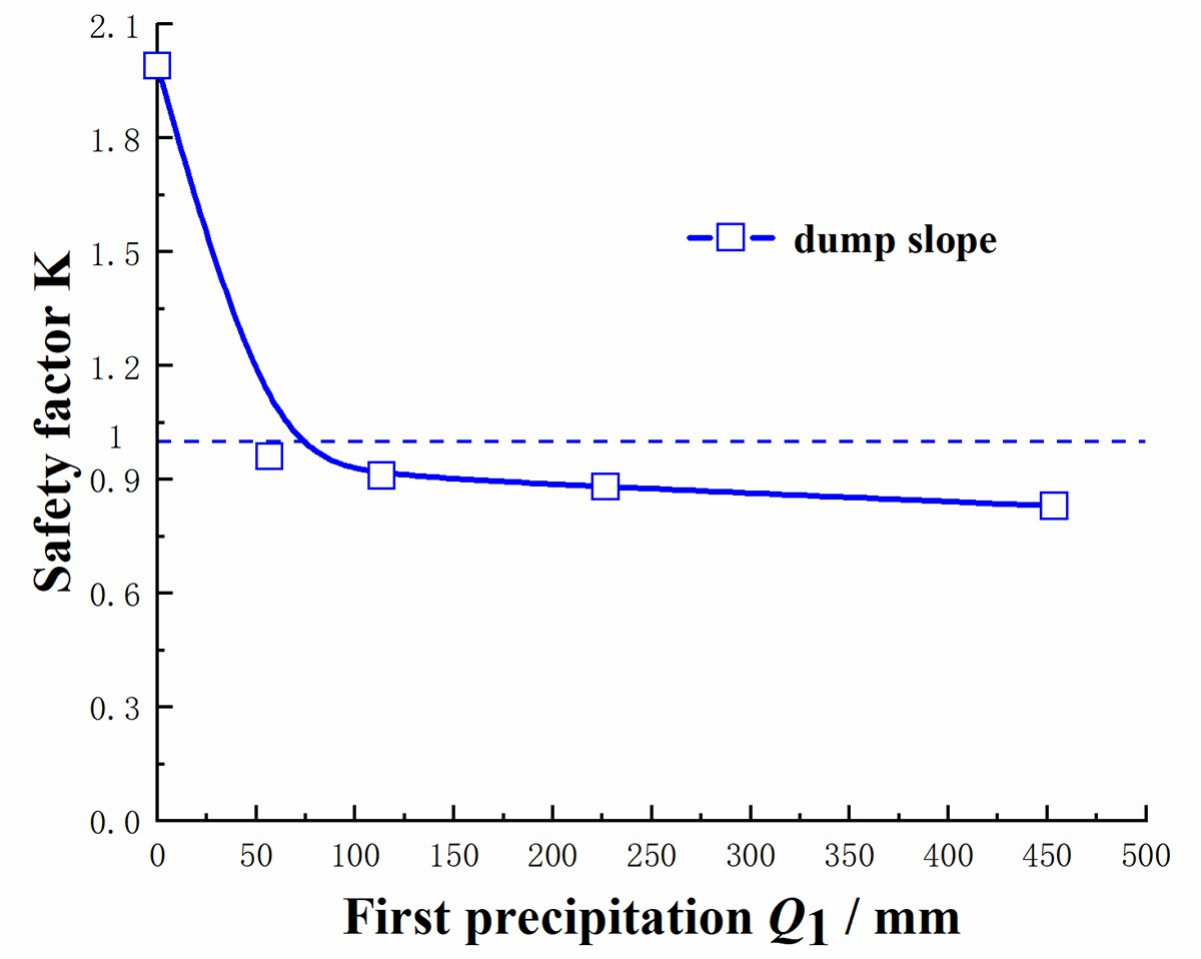
Safety factor *K* of the dump under different drying-wetting alternate intensities.

It could be found from Fig 18 that the safety factor *K* value of the dump slope in the dry season was 1.99. When the drying-wetting alternation strength was increased to 56.7mm, 113.4mm, 226.8mm and 453.6mm, the safety factor *K* value of the dump slope decreased to 0.96, 0.91, 0.88 and 0.83, respectively. From the safety factor curve of the dump slope, it could be seen that as the drying-wetting alternation intensity increased, the dump slope safety factor curve presented a monotonically decreasing trend. At *Q*_1_ ≤ 226.8 mm, the safety factor curve of the dump exhibited a steep downward tendency, and the safety factor *K* decreased significantly At *Q*_1_ > 226.8 mm, the safety factor curve of the dump showed a slow downward tendency, and the safety factor *K* value decreases slowly. It indicated that the safety factor of the dump was weakened by the influence of the drying-wetting alternation intensity. The safety factor *K* value of the dump slope under different drying-wetting alternation intensity is less than 1. According to Ref. [21], it could be seen that the slope of the dump has not met the engineering stability standard, and would be unstable and slip.

## Conclusions

Focusing on the problem of safety and stability of high-altitude dumps, which are affected by the change in the drying-wetting alternation intensity, the main conclusions are as follows:

1. As the drying-wetting alternation intensity increased, the surface soil of the dump changed from an unsaturated state to a saturated state, and the pore water pressure of the surface soil continued to increase until it reached zero, that is, the matric suction was zero, and the transient state appeared. The shear strength of the soil was weakened, the pore water pressure below the wetting front was redistributed according to the gradient, and the unsaturated soil area continued to shrink;
2. When the dump slips, the sliding area is the dump itself under the influence of different drying-wetting alternation intensities;
3. The maximum accumulated settlement of the dump exhibited a nonlinear relationship with time. With an increase in time, the maximum accumulated settlement of the dump continued to increase and finally tended to be flat. The actual monitored maximum cumulative settlement was in good agreement with the simulated maximum cumulative settlement;
4. When the drying-wetting alternation intensity increased, the safety factor of the dump decreased, and the curve showed an inverse correlation. At the low-medium-intensity and high-intensity drying-wetting alternations, the safety factor curve of the dump showed a steep downward tendency and a slow declining tendency, respectively. The safety factors of the dump under different drying-wetting alternation intensities were less than 1, indicating that the slope slipped.
